# Aphid Viruses: A Brief View of a Long History

**DOI:** 10.3389/finsc.2022.846716

**Published:** 2022-02-24

**Authors:** Ya Guo, Ning Ji, Lisha Bai, Juntian Ma, Zhaofei Li

**Affiliations:** State Key Laboratory of Crop Stress Biology for Arid Areas, Key Laboratory of Northwest Loess Plateau Crop Pest Management of Ministry of Agriculture, College of Plant Protection, Northwest A&F University, Yangling, China

**Keywords:** aphids, densovirus, bunyavirus, dicistrovirus, flavivirus, iflavirus, mesonivirus

## Abstract

Aphids are common agricultural pests with a wide range of hosts from agriculture to forestry plants. As known, aphids also serve as the major vectors to transmit plant viruses. Although numerous studies have focused on interactions between aphids and plant viruses, little is known about the aphid viruses, i.e., the insect viruses that are infectious to aphids. In the past four decades, several aphid viruses have been identified in diverse aphid species. In this review, we present a brief view of the aphid pathogenic viruses from several aspects, including classification of aphid viruses and characters of the viral genome, integration of viral sequences in host genomes, infection symptoms and influence on aphids, as well as host range and transmission modes. Taken together, these studies have increased our understanding of the rarely known aphid viruses, and will potentially contribute to the development of new strategies for controlling aphid populations.

## Introduction

Aphids belong to Hemiptera, Aphidoidea. Currently, over 5,000 species of aphids in about 500 genera have been recognized (1). The individuals of this large group of insects are widely distributed in subtropical and temperate regions of the Northern hemisphere. They have a high reproductive potential and phenotypic plasticity. Within their complex life cycles, most species adopt a seasonal periodic pathenogenesis with alternating asexual and sexual stages and generate winged (alatae) and unwinged (apterae) morphs. As polyphagous phloem-feeders, aphids are the major insect pests on a diversity of crops, such as cereals, flowers, fruits, and vegetables (2–5). During ingesting phloem sap from host plants, aphids can probe almost all plant tissues with their stylets and acquire or inoculate the virus within plants. As the most efficient vectors of plant viruses, aphids are estimated to transmit nearly over 30% of insect-borne viruses (6, 7). Additionally, aphids honeydew induce sooty molds grow on leaves and hinder the photosynthetic activities of plants (4).

For a long time, numerous studies have demonstrated the relationship of aphids and plant viruses, with emphasis on interactions between plant viruses, aphids, and plants, as well as the molecular mechanisms underlying these interactions (5, 6, 8). In contrast, insect viruses that are infectious to aphids receive little attention. In the 1960s, virus-like particles have been described in *Myzus persicae* and *Rhopalosiphum maidis* (9, 10). However, the first aphid virus, Rhopalosiphum padi virus (RhPV), has been characterized by D'Arcy et al. (11). Currently, several aphid pathogenic viruses have been identified in diverse aphid species. Based on nucleotide sequences, these viruses can be divided into two categories: (1) DNA viruses: these viruses include Dysaphis plantageinea densovirus (DplDV), Myzus persicae densovirus *(*MpDV), and Myzus persicae nicotianae densovirus (MpnDV), which are small DNA viruses and grouped into *Densovinae* in the family *Parvoviridae*; (2) RNA viruses: these viruses are further classified into different groups: (i) Bunyaviruses: including Aphid bunyavirus 1 (ABV-1) and Aphis citricidus bunyavirus (AcBV); (ii) Dicistroviruses: including Aphid lethal paralysis virus (ALPV) and RhPV; (iii) Flaviviruses: including Macrosiphum euphorbiae virus 1 (MeV-1); (iv) Iflaviviruses: including Brevicoryne brassicae virus (BrBV); (v) Mesoniviruses: including Aphis citricidus meson-like virus (AcMSV); (vi) Unclassified RNA viruses: including Aphis glycines virus 1 (ApGlV1), Aphis glycines virus 2 (ApGlV2), Acyrthosiphon pisum virus (APV), and Rosy apple aphid virus (RAAV) (Table 1). In this article, we present a brief view of the aphid viruses from several aspects, which include the taxonomy of the virus, characters of the viral genome, integration of certain virus-derived sequences in aphid genomes, infection symptoms and mechanisms, as well as host range and transmission modes.

**Table 1:** Classification and characters of aphid viruses.

**Virus category**	**Virus classification**			**Env^**&**^**	**Genome^**#**^ (GenBank no.)**	**References**
	**Family/subfamily**	**Genus**	**Virus**			
Densovirus	*Parvoviridae*/*Densovirinae*	*Ambidensovirus*	Dysaphis plantageinea densovirus	**–**	(+)ssDNA, 4979 nt (EU851411)	(12)
			Myzus persicae densovirus	**–**	(+)ssDNA, 5499 nt (AY148187)	(13)
			Myzus persicae nicotianae densovirus	**–**	(+)ssDNA, 5480 nt (KT239104)	(14)
Bunyavirus	*Phasmaviridae*	NA	Aphid bunyavirus 1	+	(**–**)ssRNA, L/M/S^*^: 7317/6626/1874 nt (NA)	(15)
	*Phenuiviridae*	NA	Aphis citricidus bunyavirus	+	(**–**)ssRNA, L/M/S^*^:7037/ 3462/1163 nt (MN163034, MN163035, MN163036)	(16)
Dicistrovirus	*Dicistroviridae*	*Cripavirus*	Aphid lethal paralysis virus	**–**	(+)ssRNA, 9812 nt (NC_004365)	(17)
			Rhopalosiphum padi virus	**–**	(+)ssRNA, 10011 nt (AF022937)	(18)
Flavivirus	*Flaviviridae*	*Flavivirus*	Macrosiphum euphorbiae virus 1	+	(+)ssRNA, 22780 nt (KT309079)	(19)
Iflavirus	*Iflaviridae*	*Iflavirus*	Brevicoryne brassicae virus	**–**	(+)ssRNA, 10161 nt (EF517277)	(20)
Mesonivirus	*Mesoniviridae*	NA	Aphis citricidus meson-like virus		(+)ssRNA, 20300 nt (MN961271)	(21)
Unclassified RNA virus	NA	NA	Acyrthosiphon pisum Virus	**–**	(+)ssRNA, 10016 nt (AF024514)	(22)
			Aphis glycines virus 1	**–**	(+)ssRNA, 8680 nt (KM015260)	(23)
			Aphis glycines virus 2	**–**	(+)ssRNA, 4850 nt (KR912180)	(24)
			Rosy apple aphid virus	**–**	(+)ssRNA, 9992 nt (DQ286292)	(12)

& *Env represents the envelope of virus*.

* *L/M/S represents the large, medium, and small segments of the virus genome*.

# *Genome: most of the virus genomes are incomplete*.

*NA, not available*.

## DNA Viruses

### Densoviruses

#### Classification and Characters of the Genome of Aphid Densoviruses

Densoviruses are non-enveloped small DNA viruses. The virus particle is icosahedral symmetry with the diameter of about 25 nm. The genome of densoviruses is single-stranded linear DNA with the length of 4–6 kb. Termini of both ends of the genome are locked with a short duplex haripin telomeres (~200 bp), which involved in DNA replication. Densoviruses contains two sets of genes encoding 2–3 non-structural (NS) and 2–4 capsid (VP) proteins (25). Currently, densoviruses are grouped into *Densovirinae* in the family *Parvoviridae*. The *Densovirinae* subfamily is further divided into five genera: *Ambidensovirus, Brevidensovirus, Hepandensovirus, Iteradensovirus*, and *Penstyldensovirus*. Ambidensoviruses have coding capacities in both directions of the strand. The NS genes are located at the 5'-proximal of the sense strand, while the VP genes are derived from the 5'-proximal of the antisense strand. In comparison, other densoviruses are monosense viruses, which encode NS proteins at the 5'-terminus, and VP proteins at the 3'-terminus of the same strand (26).

**Figure 1:**
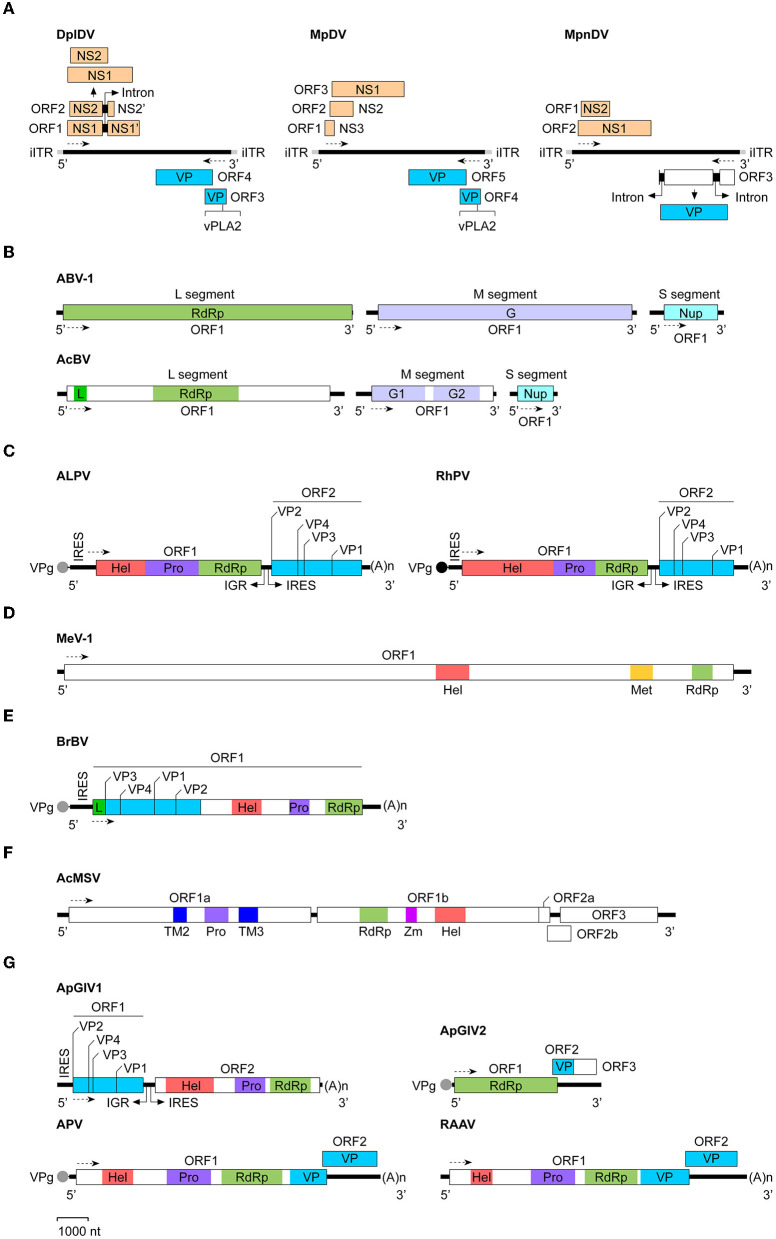
Genomic organization and expression strategies of aphid viruses. The virus genomes used were: **(A)** Densoviruses: DplDV (Dysaphis plantageinea densovirus), MpDV (Myzus persicae densovirus), and MpnDV (Myzus persicae nicotianae densovirus); **(B)** Bunyaviruses: ABV-1 (Aphid bunyavirus 1) and AcBV (Aphis citricidus bunyavirus); **(C)** Dicistroviruses: ALPV (Aphid lethal paralysis virus) and RhPV (Rhopalosiphum padi virus); **(D)** Flavivirus: MeV-1 (Macrosiphum euphorbiae virus 1); **(E)** Iflavirus: BrBV (Brevicoryne brassicae virus); **(F)** Mesonivirus: AcMSV (Aphis citricidus meson-like virus); **(G)** Unclassified RNA viruses: ApGlV1 (Aphis glycines virus 1), ApGlV2 (Aphis glycines virus 2), APV (Acyrthosiphon pisum virus), and RAAV (Rosy apple aphid virus). The GenBank accession no. of each viral genome sequence (see Table 1). The determined or putative Vpg protein at the 5′-end of the viral RNA genome is indicated as a black or gray circle. The transcription direction for each virus is showed as the dashed line with arrow. (A)n, poly(A) sequence; G, glycoprotein; G1, glycoprotein 1; G2, glycoprotein 2; Hel, RNA helicase; IGR, intergenic region; iITR, incomplete inverted terminal repeat; IRES, internal ribosomal entry site; L, L-protein; Met, methyltransferase; NS, non-structural protein; Nup, nucleoprotein; Pro, protease; RdRp, RNA-dependent RNA polymerase; TM2, transmembrane protein 2; TM3, transmembrane protein 3; VP, structural protein; VPg, virus genome-linked protein; vPLA2, virus phospholipase 2; Zm, zinc cluster-binding domain.

In 2003, van Munster et al. first identified MpDV in *M*. *persicae* (13). Like other members of *Densovirinae*, MpDV particles are isocochatral and have a diameter of ~20 nm. MpDV genome lack of the inverted terminal repeat (ITR) is 5,499 nt and predicted to encode five ORFs. Three predicted ORFs are located at the 5′-proximal region of the sense strand and encode three NS proteins (NS1-NS3) with different sizes (NS1, 698aa; NS2, 225aa; and NS3, 97aa), whereas two ORFs are located at the 5′-proximal region of the antisense strand and predicted to encode five capsid protein (VP) with the size of 57–92 kDa (13, 27) (Figure 1A). In 2009, Ryabov et al. identified DplDV in the rosy apple aphid *Dysaphis plantageinea* (12). The genome of DplDV is 4,979 nt and predicted to encode four ORFs. Two ORFs located on the 5'-proximal of the sense strand encode NS1 (710aa, encoded by ORF1) and NS2 (414 aa, encoded by ORF2), whereas the other two ORFs located on the antisense strand encode VP proteins (12) (Figure 1A). Similarly, a recently identified MpnDV genome possesses three putative ORFs with two encoding NS1 and NS2 proteins on the sense strand, as well as the third one encoding a VP protein on the antisense strand (Figure 1A). Genome sequence alignment analysis showed that MpnDV was closely related with MpDV, and both viruses may represent different strains of a same virus species (14). Based on the phylogenetic analysis and transcription assay, these aphid densoviruses are grouped in the genus *Ambidensovirus* (13, 14) (Table 1).

Additionally, partial sequence (2,668 nt) of a new densovirus Macrosiphum euphorbiae virus 2 (MeV-2) was retrieved from the transcriptome data of the potato aphid *Macrosiphum euphorbiae*. Although sequence alignment indicated that MeV-2 was closely related with DplDV, the detailed molecular characters and classification of this virus are not clear (28).

#### Integration and Transcription of Densovirus-Derived Sequences in Aphid Genomes

Retroviral and non-retroviral sequences are commonly integrated into insects, plants, and vertebrates genomes by different replication strategies (29–33). In long-term arm races between viruses and hosts, these endogenous viral elements (EVEs) either serve as the fossil record of past virus infection or play functional roles in diverse aspects in regulating host genome stability, immune response, and environmental fitness (29–31).

Aphids have remarkable wing plasticity. In response to high population densities or poor plant quality, the wing dimorphism allows asexual aphids to produce winged offspring that essentially promote aphid dispersal (2, 34). Prior studies indicated that EVEs derived from densoviruses integrated into the genome of *A*. *pisum* (35). Recently, the transcription of two EVEs (*Apns-1, Apns-2*), which showed high similarities to the NS protein sequences of MpDV and DplDV, were found to be up-regulated in response to aphid crowding in those wing highly inducible genotypes of *A*. *pisum*. RNAi knockdown the transcription of those genes substantially reduce the percentage of winged morphs of the genotype, suggesting that these densovirus-derived EVEs play critical roles in regulating wing plastic traits of pea aphids (36). Recent findings indicated that 21 desovirus-derived EVEs were integrated into 10 scaffords of the *M*. *persicae* genome (37). Although the significance of these EVEs is not clear, the relative high transcription level of some EVEs and the moderate effect of MpDV on growth and development of *M*. *persicae* suggest that the integration of desovirus-derived EVEs may regulate fitness of the virus and aphids (27, 37).

#### Infection Impact and Cytopathology of Aphid Densoviruses

Prior studies showed that the aphids (*M*. *persicae* and *D*. *plantaginea*) infected with MpDV or DplDV had a dramatic reduction of their weight, fecundity, and development time (12, 27). However, MpDV infection has no significant effect on the intrinsic growth rate of the *M*. *persicae* population (27). Intriguingly, DplDV infection led the asexual clones of *D*. *plantaginea* to produce a high percentage of winged offspring (12). This phenomenon is consistent with that observed in *A*. *pisum* that RNAi knockdown the expression of densovirus-derived EVEs impact the production of winged morphs (36). These studies suggest that densovirus infection or integration of desovirus-derived EVEs regulates the wing plasticity of aphids.

Currently, little is known about infection mechanisms of aphid densoviruses. Earlier transmission electron microscopy (TEM) studies showed that MpDV could be only replicated in the nuclei of cells of the anterior part, i.e., the stomach of the midgut of *M*. *persicae*. The nuclei of infected midgut cells usually expanded twice in size compared with that of healthy cells, and vesicles containing progeny virions were observed clustering in the perinuclear space between the inner and outer nuclear membranes. In addition, large amount of progeny virions were found in large and spherical vesicles in the cytoplasm of infected cells (27).

#### Host Range and Transmission Modes of Aphid Densoviruses

The host range of aphid densoviruses is lack of systematic investigation. Earlier studies indicated that MpDV could infect only *M*. *persicae* and possibly the whitefly *Trialeurodes vaporariorum* (family *Aleyrodidae*), but not infect the other aphids, such as *A*. *pisum, Metopolophium dirhodum, Rhopalosiphum padi*, or *Schizaphis graminum* (27).

Few studies revealed that aphid densoviruses including DplDV, MpDV, and the putative MeV-2 can be transmitted vertically from infected adults to nymphs (12, 27, 28). Interestingly, although the majority of nymphs produced by infected aphids were tested positively, a proportion of nymphs were still virus free for DplDV and MpDV (12, 27). Horizontal transmission of densoviruses from infected aphids to healthy ones was also observed in different aphids. In addition, prior studies indicated that host plants play important roles in horizontal transmission of aphid densoviruses. MpDV could be detected in healthy aphids that placed on leaves far from the infected-aphids infested leaves (12, 27, 28). Although no evidence indicate that these viruses could replicate in plants, current data suggest that aphid densoviruses may circulate in plants. The plant may serve as reservoirs for virus transmission or they may utilize the virus as a weapon defense against aphids (38).

## RNA Viruses

### Bunyaviruses

#### Classification and Characters of the Genome of Aphid Bunyaviruses

Bunyaviruses are a group of enveloped viruses that have spherical virions with the diameter of 80–120 nm. Most bunyavirus genomes consist of three segments of single-stranded, negative-sense RNA: large (L), medium (M), and small (S), which separately encodes an RdRp, envelope glycoprotein(s), and viral capsid proteins (39). Bunyaviruses can infect various hosts including invertebrates, plants, and vertebrates, and mostly are transmitted by invertebrate vectors. Since this group contains highly abundant viruses and new species are continuously found, the taxonomy of bunyavirus has been re-defined by the International Committee on Taxonomy of Viruses (ICTV) from the family *Bunyaviridae* to the order *Bunyavirales* in 2017 (40). Currently, *Bunyavirales* contains 12 families, 4 subfamilies, 54 genera and 477 species (41).

Recently, two aphid bunyaviruses, ABV-1 and AcBV were separately identified in the laboratory cultured aphids *A*. *pisum* or *Aphis citricidus*. The genome sequences of both viruses retrieved from the transcriptome data of the aphids were single-stranded, negative-sense RNA that composed of three segments: L (ABV-1, 7,317 nt; AcBV, 7,037 nt), M (ABV-1, 6,626; AcBV, 3,462 nt), and S (ABV-1, 1,874 nt; AcBV, 1,163 nt) (Table 1). Each segment of the virus genome contains one ORF, which separately encodes an RdRp (ABV-1) or an RdRp and an L-protein (AcBV) (L segment), the envelope glycoprotein(s) (M segment), and the nucleoprotein (S segment) (Figure 1B). Phylogenetic analysis of the RdRp domain indicated that ABV-1 and AcBV are grouped to the family *Phasmaviridae* or *Phenuiviridae*, respectively (15, 16).

#### Host Range and Transmission of Aphid Bunyaviruses

Prior studies indicated that ABV-1 could infect a bunch of aphids, such as *Aphis aurantii, A*. *citricidus, A*. *gossypii, A*. *pisum, A*. *spiraecola, M*. *persicae, R*. *padi, Rhodobium porosum*, and *S*. *avenae*. This virus may be transmitted vertically from infected aphids to nymphs or horizontally via plants infested by infected aphids (15).

### Dicistroviruses

#### Classification and Characters of the Genome of Aphid Dicistroviruses

Dicistroviruses belong to the family *Dicistroviridae* (42). Single-stranded positive-sense RNA genome with the length of 9–11 kb of these viruses are packaged in non-enveloped virion particles (25–30 nm in diameter). The 5'-terminus of the genome is covalently linked to a virus-encoded small protein VPg (virus genome-linked protein) and the 3'-terminus is polyadenylated. In general, dicistroviruses contain two ORFs that connected by a short intergenic region (IGR). The ORF1 located on the 5'-proximal of the genome encodes the NS proteins: an RNA helicase, a cysteine protease, and the RNA-dependent RNA polymerase (RdRp), whereas ORF2 located on the 3'-proximal of the genome encodes a large polyprotein which is processed to generate VP1-VP4 structural proteins. In addition to these features, dicistroviruses also contain an internal ribosome entry site (IRES) in front of each ORF that required for directing translation initiation in an AUG-independent manner (43, 44).

Dicistroviruses can infect various arthropods, including the insects in diptera, hemiptera, hymenoptera, and orthoptera (44). Currently, only two aphid discistroviruses ALPV and RhPV were characterized. Both of the viruses were identified in *R*. *padi* many years ago and grouped with *Drosophila C virus* and *cricket paralysis virus* in the genus *Cripavirus* of *Dicistroviridae* (Table 1) (11, 18, 45, 46). Similar with the other member of *Dicistroviridae*, the positive-sense RNA genome of aphid dicistroviruses with the length of 9,812 nt (ALPV) or 10,011 nt (RhPV) are packaged in about 26 nm virion particles. The NS proteins encoded by ALPV or RhPV ORF1 contian an RNA helicase, a cysteine protease, and an RdRp domains, whereas the protein precursor encoded by ORF2 may be cleaved to form four structural proteins (VP1-VP4) (Figure 1C) (18, 46). Recently, different isolates of ALPV has been identified in diverse insects including aphids (*Aphis nerii, A*. *fabae, A*. *pisum, R*. *padi*) (17, 47, 48), bees (*Apis mellifera, Apis cerana*, and *Verpa velutina*) (49–52), and beetles (*Diabrotica virgifera*) (17), in bats (*Hipposideros caffer*) (53), and in plants (54). Genome sequences analysis indicated that ALPV isolates obtained from *A*. *pisum* and *A*. *mellifera* repectively are closely related, but both of them have relatively low sequence identities with those isolated from other insects and bats, suggesting that the isolates of ALPV from different arthropods may represent distinct ALPV strains or species (17).

#### Infection Symptoms and Mechanisms of Aphid Dicistroviruses

Under laboratory conditions, infection of ALPV and RhPV usually causes substantially distinct pathological symptoms in different aphids. For *R. padi*, the virus infection results in a short lifespan of the aphids, significant reduction of the fecundity, high mortality of nymphs, and a substantial decrease of population density (55, 56). In addition, ALPV infection also causes uncoordinated movement and paralysis of *R. padi* (45). Interestingly, RhPV infection was found to affect olfactory behavior of *R. padi* and cause infected aphids easily be predated or attacked by natural enemies (57). In contrast, an ALPV isolate identified from the wild population of the milkweed aphid *A*. *nerii* in northern Israel is asymptomatic in *A. nerii*, however, the virus could infect *M. persicae* and cause high mortality of the aphid (48). In an earlier field investigation, Laubscher and Von Wechmar found that a dramatic decline in grain aphids *R*. *padi* and *S*. *avenae* numbers were coincided with the high incidence of ALPV present in the aphid population, suggesting that ALPV could serve as a population growth-limiting factor in controlling aphids (56).

Current knowledge of infection mechanisms of aphid dicistroviruses is very limited. Earlier TEM analysis revealed that RhPV infection occurs in the posterior midgut cells or hindgut cells of infected aphids, and the infection was not observed in the anterior midgut (stomach), salivary gland, tracheal, nerve or muscle cells. Within the gut cells, RhPV replicates in the cytoplasm and the infection leads to the absence of the cytoplasmic organelles, except for mitochondria. Virions were found free or packed in crystalline arrays in large vesicles in the cytoplasm (58). Similar cytopathic effect (CPE) was also observed in RhPV-infected GWSS-Z10 cells (59), which are derived from the embryos of *Homalodisca coagulate* (60). Since the mitochondria and nuclear membranes remained intact during infection and the virus particles were not observed in the nuclei, these data imply that RhPV may replicate and assemble in the gut cells mitochondria.

#### Host Range and Transmission Modes of Aphid Dicistroviruses

Dicistroviruses have a variety of hosts (44). Earlier studies have demonstrated that RhPV could infect a narrow range of economically important grain aphids, including *A*. *dirrhodum, Diuraphis noxia, R*. *maidis, R*. *padi, R*. *rufiabdominalis*, and *S*. *graminum* (55). In contrast, the host range of ALPV is complicated. This virus was found to infect several distinct aphid species, such as *A*. *nerii, M*. *dirhodum, M*. *persicae, R*. *padi, S*. *avenae*, and *S*. *graminum*, and the whitefly *T*. *vaporatiorum* (48, 58, 61). In addition, as mentioned above, deep-sequencing analysis indicated that ALPV or ALPV-like viruses were present in other insects including bees and beetles, in bats, and in plants (17, 54, 62). Although the infection and pathogenecity of the virus in those animals and plants remain unclear, the high sequence similarity among different ALPV isolates suggest that ALPV-like viruses may have a wide range of hosts.

RhPV can actively spread among different individuals of aphids and plants play an important role in mediating the virus horizontal transmission. It was found that RhPV was present in all parts of the plant which were infested by infected aphids (63). Although RhPV seems to replicate less likely in plants, host plants may act as a virus reservoir and promote virus circulating in the aphid population (63, 64). Also, the virus may temporarily exist in plants during the winter season when aphids are absent in the environment. RhPV can be transmitted vertically from adult aphids to nymphs and the virus has been detected in different life stages of *R*. *padi*, including sexual aphids and eggs (55, 63). However, the transovarial passage rate of the virus was found to be substantially lower than that in horizontal transmission (55), which might be caused by the lower survival rate of nymphs produced by infected aphids.

### Flaviviruses

#### Classification and Characters of the Genome of Aphid Flaviviruses

Flaviviruses are grouped in the genus *flavivirus* in the family *Flaviviridae*. The single-stranded positive-sense RNA genome, 9.2–11 kb in length, is assembled in small, enveloped virions (about 50 nm in diameter). The 5'-end of the genome is blocked with an m-7GpppAmp cap and the 3'-end is lack of a polyadenylated tail. Flaviviruses possess a long ORF that encodes a polyprotein, which is subsequently processed to generate structural and non-structural proteins. Structural proteins (VPs) are located at the N-proximal proportion of the polyprotein, whereas NS proteins including an RNA helicase, a serine protease, and the RdRp are located at the C-terminal region (65).

Recently, a putative aphid flavivirus, named as Macrosiphum euphorbiae virus 1 (MeV-1), was identified in the potato aphid *Macrosiphum euphorbiae*. The genome of MeV-1 retrieved from the transcriptome data of *M*. *euphorbiae* is 22,780 nt and contains a single large ORF encoding a polyprotein of 7,333 amino acids. Homology analysis indicated that the polyprotein of MeV-1 contains the helicase, methyltransferase, and RdRp domains (Figure 1D). Phylogenetic analysis of the helicase or RdRp domain of MeV-1 indicated that this virus belongs to *Flaviviridae*. The presence of the methyltransferase domain in the polyprotein indicates that MeV-1 is a putative member of the genus *Flavirivus* (Table 1) (19).

#### Infection Symptoms and Transmission of Aphid Flaviviruses

No obvious infection symptoms or abnormal phenotypes were observed in MeV-1 infected *M*. *euphorbiae*. Viral RNA and its replication intermediates were detected in all developmental stages of aphids and all newborn nymphs collected after laid off and grew on natural tomato plants, and the virus was not integrated into the *M. euphorbiae* genome, suggesting that MeV-1 can be transmitted vertically. In addition, MeV-1 was detected in aphids-infested plant leaves, but the virus titer reduced dramatically after removal of the aphids, indicating that the virus could not replicate in plants and the presence of the virus in plants may contribute to the horizontal transmission of MeV-1 (19).

### Iflaviruses

#### Classification and Characters of the Genome of Aphid Iflaviruses

Iflaviruses, a group of non-enveloped, small RNA viruses with the virion size ranging from 20 to 30 nm in diameter, belong to the family *Iflaviridae*. The single-stranded positive-sense RNA genome with the length of 8–10 kb contains a single ORF, which encodes a polyprotein that is subsequently processed to yield NS and VP proteins. NS proteins that generated by the C-terminal region of the polyprotein function as an RNA helicase, a 3C-like cysteine protease, and an RdRp. Four VP proteins (VP1-VP4) produced by the N-terminal region of the polyprotein coordinate to form the virion capsid. Iflaviruses replicate in the cytoplasm of host cells, but the molecular mechanism of the virus infection remain unknown yet (66).

Currently, there is only one aphid iflavirus, Brevicoryne brassicae virus (BrBV), was identified from the infected cabbage aphid *Brevicoryne brassicae* (Table 1) (20). The single-stranded positive-sense RNA genome of BrBV, 10,161 nt in length, contains a single ORF, which is predicted to encode a polyprotein of 2,983 amino acids. Sequence alignments indicated that the N-terminal region of BrBV polyprotein has high similarities with the VP proteins of other iflaviruses, whereas the C-terminal region of the polyprotein contains distinct regions of sequence identical to the RNA helicase, a chymotrypsin-like protease, and an RdRp (Figure 1E). Phylogenetic analysis revealed that BrBV is grouped together with *Dinocampus coccinellae paralysis virus* (20). Recently, a new isolate of BrBV (BrBV-IL) has been identified in aphids *B*. *brassicae* collected from wild mustard plants *Sinapis arvensis* in Israel. BrBV-IL genome shares a 95% nucleotide sequence identity with that of BrBV (67).

#### Infection Mechanisms of Aphid Iflaviruses

To date, infection mechanisms of BrBV remain unclear, but the virus may not persist or replicate in *Brassica* and *Arabidopsis* plants. Intriguingly, the natural population of *B*. *brassicae* with a heavy infeciton of BrBV was less parasitized by parasitoid wasps, implying that BrBV infection affects the interaction between *B*. *brassicae* and its natural enemies (20).

### Mesoniviruses

#### Classification and Characters of the Genome of Aphid Mesoniviruses

Mesoniviruses, a group of enveloped, single-stranded positive-sense RNA viruses that classified into the family *Mesoniviridae*, have a genome of ~20 kb that contains 6–7 ORFs. Two large overlapped ORFs (ORF1a and ORF1b) located on the 5'-proximal of the genome (~15 kb) encodes the NS proteins, which are arranged in order as the 3C-like protease (3CLpro, encoded by ORF1a), RdRp, zinc-binding module and associated helicase (ZnHel1), exoribonuclease (ExoN), N^7^-methyltransferase (NMT), and 2'-*O*-methytransferase (OMT) (encoded by ORF1b). The other ORFs located at the 3'-terminus of the genome encode the structural proteins, including the spike (S) protein and other membrane proteins (68). Mesoniviruses have been isolated from infected mosquitoes and these viruses seems non-infectious to vertebrates (68, 69).

Recently, a new mesonivirus, AcMSV, has been identified in the brown citrus aphid *A*. *citricidus* (21). The viral genome retrieved from the transcriptome data of *A*. *citricidus*, 20,300 nt in length, contains five ORFs encoding six conserved proteins, including transmembrane protein 2 (TM2), a protease, TM3, an RdRp, a zinc cluster-binding domain (Zm), and an RNA helicase (Figure 1F). Phylogenetic analysis using the RdRp domain of AcMSV showed that this virus belongs to *Mesoniviridae* (Table 1) (21).

### Unclassified RNA Viruses

#### Acyrthosiphon Pisum Virus

##### Classification and Characters of the Genome of APV

APV is a positive-sense, single-stranded RNA virus with the genome of 10,016 nt (Table 1). The virion is about 31 nm in diameter and composed of four VP proteins with the molecular weight of 23.3, 24.2, 34.5, and 66.2 kDa. Sequence analysis indicated that APV genome contains two ORFs. The large ORF1 (7,890 nt) is located on the 5'-proximal of the genome and predicted to encode a polyprotein of 296.3 kDa, which possesses the picorna-like RNA helicase, chymotrypsin-like cysteine protease, and RdRp domains. The small ORF2 (1,665 nt) is located at the 3'-end of the genome and overlaps the ORF1 by 28 nt. It's predicted to encode a protein of 63.3 kDa. Amino acid sequencing analysis showed that the capsid proteins of APV are produced by the 3'-half of the genome (Figure 1G). They may encode by the 3'-end of ORF1 (34.5 kDa) or ORF1 and ORF2 (66.2 kDa), or generate by the proteolytic cleavage of the 34.5 kDa protein (23.3 and 24.2 kDa). In addition, a subgenomic RNA of ~4 kb was detected in both purified APV and the virus-infected aphids (22).

##### Infection Impact, Cytopathology, and Transmission of APV

Although APV infection inhibited *A*. *pisum* nymphs growth and prolonged the development time (70), recent studies suggested that the virus infection may play a beneficial role in mediating adaption of aphids to low-fitness plants (71). During infection, APV was predominantly present in salivary glands and the gut epithelial cells of *A*. *pisum* nymphs (70, 71), but also observed in muscle cells and mycetocytes (70). It seems that the virus replicates in the cytoplasm and virions may be clustered in small vesicles. APV could not replicate in plants, but it can be persisted in plants for a few days and allowed horizontal transmission to aphids. The virus was found to be vertically transmitted to offspring of *A*. *pisum*. However, the transovarial transmission rate of APV was substantially low, which might be caused by the low level of the virus in ovaries of adult aphids (70).

#### Aphis Glycines Virus 1

ApGlV1 was identified in the field population of *A*. *glycines*. The viral genome is a single-stranded bicistronic RNA with the length of 8,680 nt (excluding the 3'-end polyadenylated tail) (Table 1). The genome contains two large ORFs, which are separated by an IGR fragment of 474 nt. In front of each ORF, there is an IRES element required for translation. ORF1 located on the 5'-proximal of the genome encodes four VP proteins, whereas the 3'-proximal ORF2 encodes a polyprotein containing an RNA helicase, a protease, and an RdRp domains (Figure 1G). The genomic structure of ApGlV1 is similar with that of dicipiviruses in the family *Picornaviridae*. Phylogenetic analysis indicated that ApGlV1 is grouped with the unclassified picorna-like viruses (23).

#### Aphis Glycines Virus 2

ApGlV2 was identified in the laboratory cultured *A*. *glycines*. ApGlV2 genome is a single-stranded positive-sense RNA with the length of about 4,850 nt (Table 1), which contains two predicted overlapping ORFs. ORF1 encodes a putative replicase of 1,121 amino acids, possessing the RdRp domain. ORF2 is predicted to encode a capsid protein (P24). In addition, at the downstream of the coding region of P24, there may exist an additional ORF3 which encodes a protein with the unknown function (Figure 1G). Although the RdRp domain of ApGlV2 showed low amino acid sequence similarities with the other RNA viruses, structure analysis suggested that the predicted tertiary structure of ApGlV2 RdRp was similar to those of tetraviruses and Drosophila A virus. ApGlV2 is not integrated into the genome of *A*. *glycines*. The virus could be transmitted vertically, but the horizontal transmission mode remain unknown yet (24).

#### Rosy Apple Aphid Virus

##### Classification and Characters of the Genome of RAAV

RAAV was detected in *D*. *plantageinea*. Virion particles of this virus are about 32 nm in diameter. The viral genome is a single-stranded positive-sense RNA with the length of 9,992 nt (Table 1). Similar with that of APV, RAAV contains two ORFs. The 5′-proximal large ORF1 (7,956 nt) is predicted to encode a polyprotein possessing the picorna-like RNA helicase, chymotrypsin-like cysteine protease, and RdRp domains. Additionally, the 3′-end of ORF1 may produce a small capsid protein. The 3′-proximal small ORF2 (1,692 nt) is overlapped the ORF1 by 54 nt and predicted to encode another capsid protein (Figure 1G). RAAV has a high nucleotide sequence identity with APV.

##### Transmission of RAAV

The virus can be vertically transmitted to offspring of the aphid, but the transmission rate was found to be extremely low. In addition, virus-free aphids that placed on leaves far from the infected-aphids contaminated leaves became RAAV positive, suggesting that this virus could be horizontally transmitted via plants (12).

## Conclusion and Perspectives

In their complex life cycles, aphids, like other animals, are widely attacked by viruses. Compared with the other insect viruses, such as the extensively studied baculoviruses, the knowledge and research system of aphid pathogenic viruses are limited.

In recent years, next-generation sequencing and metagenomic analysis accelerate the finding of new aphid viruses (15, 21, 50, 62). Virus mining using viral transcripts or genomic sequences retrieved from the aphid genome or transcriptome data can save time spent on isolating or purifying virus particles, which usually need to collect a large scale of infected aphids. In addition, deep-sequencing analysis is profoundly useful for identifying viruses that cause a chronic infection and/or atypical symptoms in aphids and evaluating integration of viral elements into aphid genomes.

Although a few aphid viruses, such as ALPV, MpDV, and RhPV, showed dramatic negative impact on aphids growth or development, there is no application of these viruses as biopesticides for aphid control. A limitation is the obstacle to produce large quantities of virus. Using other insect viruses, such as the baculovirus expression system, or deleloping permissive cell lines to produce aphid viruses will be useful for large-scale virus production. Alternatively, generating transgenic plants to express aphid viruses may promote host plants defensing against aphids. In addition, infection of RhPV was found to be beneficial to the predation or attack of natural enemies by affecting the olfactory behavior of aphids *R. padi* (57), this implies that the use of aphid viruses may enhance the control effect of natural enemies on aphids.

Currently, viral infection mechanisms and effects of the virus on field population dynamics of aphids are largely unknown. Intriguingly, prior studies on aphid densoviruses and APV suggested that the virus infection or the virus-derived sequences integrated in certain aphid genomes play important roles in regulating aphids development and dispersal, or promoting aphids adaption to low-fitness plants (12, 36, 71). These pioneering studies will facilitate future investigation of the complex interaction of aphid viruses, aphids and the host plant. In addition, *in vitro* cultured cell lines of aphids are urgent to characterize the viral genome replication, gene function and virus particles assembly. It is worth noting that, although some aphid viruses, such as ALPV and RhPV, cause acute infection in certain aphids (17, 55, 61), they have been found in other animals, including bees, bettles, and bats, or in plants (17, 50, 53, 54), raising a question about the host range and pathogenecity of these and other aphid viruses. Taken together, the systematic study of aphid viruses will not only provide new strategies for controlling aphids population, but also contribute to the protection of beneficial organisms including bees and other invertebrates.

## Author Contributions

YG and ZL drafted the manuscript. All authors contributed to revising the manuscript. All authors contributed to the article and approved the submitted version.

## Funding

This work was supported by Chinese Universities Scientific Fund (Z1090121096, NWAFU) and the grant from National Key R&D Program of China (2017YFC1200605).

## Conflict of Interest

The authors declare that the research was conducted in the absence of any commercial or financial relationships that could be construed as a potential conflict of interest.

## Publisher's Note

All claims expressed in this article are solely those of the authors and do not necessarily represent those of their affiliated organizations, or those of the publisher, the editors and the reviewers. Any product that may be evaluated in this article, or claim that may be made by its manufacturer, is not guaranteed or endorsed by the publisher.
